# The Dark That Matters: Long Non-coding RNAs as Master Regulators of Cellular Metabolism in Non-communicable Diseases

**DOI:** 10.3389/fphys.2019.00369

**Published:** 2019-05-22

**Authors:** Alessia Mongelli, Fabio Martelli, Antonella Farsetti, Carlo Gaetano

**Affiliations:** ^1^Laboratory of Epigenetics, ICS Maugeri S.p.A., Pavia, Italy; ^2^Molecular Cardiology Laboratory, IRCCS Policlinico San Donato, Milan, Italy; ^3^Institute of Cell Biology and Neurobiology, National Research Council, Università Cattolica di Roma, Rome, Italy

**Keywords:** lncRNA, lipids, carbohydrates, amino acids, metabolism, cancer, diabetes, cardiovascular

## Abstract

Non-coding RNAs are pivotal for many cellular functions, such as splicing, gene regulation, chromosome structure, and hormone-like activity. Here, we will report about the biology and the general molecular mechanisms associated with long non-coding RNAs (lncRNAs), a class of >200 nucleotides-long ribonucleic acid sequences, and their role in chronic non-transmissible diseases. In particular, we will summarize knowledge about some of the best-characterized lncRNAs, such as H19 and MALAT1, and how they regulate carbohydrate and lipid metabolism as well as protein synthesis and degradation. Evidence is discussed about how lncRNAs expression might affect cellular and organismal metabolism and whether their modulation could provide ground for the development of innovative treatments.

## Introduction

Non-coding RNAs (ncRNAs) are a class of ribonucleic acid sequences which do not carry any information for protein translation, at least not for long peptides, but are demonstrably involved in gene and protein regulation (Monticelli et al., 2005), RNA splicing (Kishore and Stamm, 2006), chromosome structure (Park et al., 2002) or, traveling through circulating blood, they play some hormone-like activities (Knoll et al., 2015). Their length principally classifies ncRNAs. Conventionally, 200 nucleotides (nts) are the cut-off length determining to which group they belong to: long non-coding RNAs (lncRNAs) are longer than 200 nts, while small ncRNAs (sncRNA) are much shorter (Pedersen et al., 2006). Interestingly, although not widely used, an alternate classification has been proposed based on ncRNAs biological function, such as gene silencing or transcriptional activation, which might result much more informative and helpful for translational and clinical investigations (Amaral et al., 2011).

Regarding lncRNAs, they include: imprinted lncRNAs, which control expression of imprinted genes (Sleutels et al., 2002); disease-associated lncRNAs which are particularly abundant in pathophysiological conditions (Matouk et al., 2007; Yoshimizu et al., 2008); pathogen-induced lncRNAs which are produced and modulated by exogenous microorganisms (Zhou et al., 2016); bifunctional RNAs that can have more than one role and can also be translated into proteins (Hashimoto et al., 2009); miRNA sponges that interfere with the inhibiting activity of miRNAs (Chen L. et al., 2018; Zhao et al., 2018). Despite a growing body of knowledge about lncRNA regulation and function, it must be said that most lncRNAs identified so far are yet “unknown” being physiologically transcribed but with a mostly undetermined function.

Evidence supports the presence of circular RNAs (circRNAs) which are 500 nucleotides on average (Salzman et al., 2012). Many processes can produce circRNAs; an example is the co-transcription of pre-mRNAs (Zhang et al., 2016) or post-transcriptional back splicing in which the exon excised contains lariat that is not connected to pre-mRNA (Suzuki et al., 2006; Barrett et al., 2015). These differences in circRNA biogenesis might be significant for their biological and pathophysiological properties. Co-transcribed circRNAs, modulate linear mRNA directly in *cis* (Ashwal-Fluss et al., 2014), while post-transcriptionally generated circRNAs do not (Barrett and Salzman, 2016).

Another class of lncRNAs is the large intergenic ncRNAs (lincRNAs) which, at least in part, controls gene expression recruiting enzymes involved in histone modifications (Khalil et al., 2009). It has been shown that dysregulations of lincRNAs are associated with epigenetic modifications occurring in cancer progression and metastasis (Gupta et al., 2010).

However, lncRNAs share many of their regulatory features with normal coding messenger RNAs (mRNA). Similar to mRNAs lncRNAs are transcribed by RNA Polymerase type II, indeed lncRNA promoters are enriched in histone H3 lysine-4 tri-methylated (H3K4me3) while, along the gene body, tri-methylation is enriched on histone H3 lysine-36 (H3K36me3) residues, a feature that allows RNA Polymerase II to start transcription (Guttman et al., 2009). Besides, lncRNAs undergo post-transcriptional modifications such as splicing, polyadenylation and 5′-capping (Sas-Chen et al., 2017) and their 3′UTR is organized similarly to that of mRNAs (Niazi and Valadkhan, 2012). Nevertheless, lncRNAs have also unique traits: (i) the nucleus is the subcellular compartment in which they are particularly abundant (Cabili et al., 2015); (ii) surprisingly enough, they may have small open reading frames (ORFs) which are sometimes converted in very short peptides of still unknown function (Ma et al., 2013; Aboudehen et al., 2018); (iii) although their abundance is physiologically lower than that of mRNAs, lncRNAs are more cell- or tissue-specific than coding mRNAs (Zimmer et al., 1994).

This review is focused on the pathophysiological relevance of lncRNA associated to, cancer and cardiovascular diseases with special attention paid to those processes arising from dysmetabolic conditions. Also, particular emphasis is given to those lncRNAs for which potential therapeutic and diagnostic applications have been envisaged. A broad description of ncRNA role across a larger number of non-communicable diseases has been provided elsewhere (Ma et al., 2013; Greco et al., 2015; Barrett and Salzman, 2016; Viereck and Thum, 2017; Rafiee et al., 2018) and is beyond the scope of this article.

## LncRNAs and Non-Communicable Diseases: An Overview

Cancer is probably the most common non-communicable disease in which important prognostic/therapeutic applications are envisaged for lncRNAs. An example of this perspective is given by the actin filament associated protein one antisense RNA1 (AFAP1-AS1) which is up-regulated in breast cancer. Its expression levels correlate well with poor prognosis (Ma et al., 2018). Similarly, high levels of AFAP1-AS1, in colon cancer, have been associated with malignancy (Tang et al., 2018), suggesting an oncogenic role for this lncRNA although the molecular mechanism associated with this property is unclear. On the opposite, the lncRNA-downregulated in liver cancer stem cells (DILC) is down-regulated in colorectal cancer where it has a tumor suppressor function. DILC interferes with the IL-6/STAT3 pathway inhibiting cell growth and metastasis (Gu et al., 2018). Another lncRNA, which has an anticancer function, is named zinc finger antisense 1 (ZFAS1). ZFAS1 is down-regulated in breast cancer, and increasing ZFAS1 level may cause cell cycle arrest and apoptosis (Fan et al., 2018). MALAT1 is probably the best-characterized lncRNAs in cancer biology. In fact, in colorectal cancer high levels of MALAT1 have been associated with increased proliferation and migration (Xu et al., 2018), whereas in oesophageal cancer, it promotes epithelial-to-mesenchymal transition through the modulation of Notch pathway (Chen M. et al., 2018). In addition, high levels of MALAT1 confer resistance to pharmacological treatment, such as cisplatin in lung cancer (Fang et al., 2018). Finally, in ovarian cancer MALAT1 interferes with the Notch pathway conferring chemo-resistance (Bai et al., 2018) (see Table 1).

**Table 1:** Expressions and effects of lncRNAs in most common cancers.

LncRNA	Expression	Disease	Role	References
AFAP1-AS1	Up-regulated	Breast cancer	Unknown	Ma et al., 2018
	Up-regulated	Colon cancer	Unknown	Tang et al., 2018
DILC	Down-regulated	Colon cancer	Interferes with IL-6/STAT3 pathway	Gu et al., 2018
ZAFAS1	Down-regulated	Breast cancer	Unknown	Fan et al., 2018
MALAT1	Up-regulated	Colon cancer	Unknown	Xu et al., 2018
	Up-regulated	Breast cancer	Interferes with Notch pathway	Bai et al., 2018
	Up-regulated	Lung cancer	Interferes with Notch pathway	Fang et al., 2018


Nowadays, obstacles in treating cancer *via* modulation of lncRNA expression apparently arise from cancer heterogeneity and genomic instability. It remains, however, an exciting challenge. A more extensive overview of the role of lncRNAs in cancer is out of the scope of this article while it has been the object of more extensive discussion in other recent reviews (Rafiee et al., 2018; Sanchez Calle et al., 2018).

Although probably less characterized than in cancer, the RNA level of different classes of ncRNAs changes significantly in CVD, including miRNAs, circRNAs, and lncRNAs (Viereck and Thum, 2017). Interestingly, a specific lncRNAs signature, composed of six lncRNAs: uc010yfd.1, RNA147299| p0403_imsncRNA819, ENST00000444488.1, ASO3973, ENST00000602558.1, and ENST00000561165.1, has been recently identified in myocardial infarction (MI) and proposed as a sensitive biomarker of coronary disease (Li L. et al., 2018). However, in this pathophysiological condition, also the cancer-associated lncRNA MALAT1 has been proposed to be of potential relevance, being highly expressed in the peripheral blood of infarcted mice (Hu et al., 2018) as a possible consequence of endogenous activation of the hypoxia pathway (Choudhry and Mole, 2016). MALAT1 might act as a molecular sponge for microRNAs, including miR-320, promoting apoptosis in infarcted cardiomyocytes (Hu et al., 2018). Cardiac hypertrophy is another situation in which lncRNAs may play a role. High levels of the cardiac-hypertrophy-related factor (CHRF) lncRNA have been found associated with this pathophysiological condition. This lncRNA sequesters miR-489, known to inhibit the mRNA encoding for the myeloid differentiation primary response gene product 88 (Myd88), increasing cardiomyocyte volume (Wang et al., 2014). On the opposite, the low levels of myosin heavy chain (MHC) associated RNA transcript lncRNAs (Mhrt) have been found associated with cardiac hypertrophy. Indeed, Mhrt binds Brg1, an ATP-dependent chromatin remodeling factor, inhibiting Brg1/DNA interaction (Han et al., 2014). As a result, pro-hypertrophic genes are not efficiently transcribed (Han et al., 2014).

In most of the cases, the molecular mechanisms associated with lncRNAs in cardiovascular accidents, heart failure, atherosclerosis, and ischemia is still unknown (Thum and Condorelli, 2015; Chatterjee et al., 2017). It must be said, however, that the multi-layered regulation of lncRNAs expression and function does not facilitate gathering mechanistic insights. For example, the long non-coding/circRNA named ANRIL has a role in CVD and diabetes. In this case, disease conditions have been associated with some single-nucleotide polymorphisms (SNPs) present in its genomic sequence at 9p21 region which is close to CDKN2A and CDKN2B tumor suppressor genes (Helgadottir et al., 2007; Wei et al., 2015). Specifically, in patients with MI, it has been found a significant presence of ANRIL genomic locus variants such as rs10757278 (Helgadottir et al., 2007), rs10965215 and rs10738605 (Cheng et al., 2017), highlighting the concept that MI predisposition might be associated with the genetic variation of an epigenetic non-coding modulator. Moreover, with this evidence, in diabetic patients, other ANRIL SNPs have been involved in cardiovascular illnesses such as coronary artery disease (CAD). T2D patients that carry SNPs rs2891168 have a higher risk to develop CAD compared to controls (Broadbent et al., 2008). Consistently, rs10757274 and rs1333042 SNPs are common in Iranians which suffers from CAD (Mafi Golchin et al., 2017). Also, increased levels of ANRIL have been observed in left ventricle biopsies derived from ischemic heart failure patients (Greco et al., 2016). However, ANRIL exists also in circular form (circANRIL). Recently, circANRIL has been found involved in atherosclerosis because of its function as ribosomal RNA maturation modulator (Holdt et al., 2016). In fact, in vascular smooth muscle cells and macrophages, circANRIL binds pescadillo homolog 1 (PES1), a 60S-preribosomal assembly factor (Holdt et al., 2016). As a result, cells undergo growth arrest and apoptosis a condition that might reduce atherosclerotic plaques formation by culling hyper-proliferating cell types in atherosclerotic plaques (Holdt et al., 2016). Indeed, carriers of a coronary artery disease-protective haplotype displayed increased expression of circANRIL. Importantly, the effect of circANRIL was inverse to that of its linear counterpart (linANRIL), which is downregulated in patients carrying the 9p21 protective genotype (Holdt et al., 2013). Accordingly, the highest circANRIL/linANRIL ratio was found in patients free of coronary artery disease.

Additional data obtained in a rat model of atherosclerosis stress the importance of the techniques used to modulate circANRIL *in vivo* and of the targeted cells (endothelial cells vs. smooth muscle and macrophages). Indeed, in this rat model, reduced circANRIL in vascular endothelial cells has been correlated with decreased inflammatory factors expression, reduced coronary atherosclerosis, and apoptosis, as well as lower endothelial damage (Song et al., 2017). The lncRNA H19 has been demonstrated to exacerbate cardiac hypertrophy (Liu L. et al., 2016) and atherosclerosis (Pan, 2017; Zhang et al., 2017; Bitarafan et al., 2019). In mouse model has been shown that miR-675, a target of H19, down-regulates CAMKIIδ expression (Liu L. et al., 2016) which is a kinase that induces cardiac hypertrophy (Anderson et al., 2011). The overexpression of H19 leads the transduction of CAMKIIδ increasing cardiomyocyte volume (Liu L. et al., 2016). High levels of H19 have been found in serum patients with atherosclerosis (Pan, 2017), indeed, in mouse vascular smooth muscle cells, upon up-regulation of H19, low level of caspase 3 has been found while proliferation is improved probably due to the activation of MAP-ERK pathway linked to inflammation (Pan, 2017). Associated to H19, in atherosclerotic patients high plasma levels of long intergenic ncRNA predicting cardiac remodeling (LIPCAR) has been observed (Zhang et al., 2017) suggesting these two lncRNAs as novel biomarkers of CVD. However, LIPCAR’s role in the development of the plaque remains unclear. Moreover, the up-regulation of H19 have been reported in peripheral blood mononuclear cells (PBMCs) of coronary artery disease patients but the role of this lncRNA and the molecular mechanisms altered in these cells are still unknown (Bitarafan et al., 2019) (see Table 2).

**Table 2:** LncRNA and CVD.

LncRNA	Expression	Disease	Role	References
MALAT1	Up-regulated	MI	miRNAs sponge	Hu et al., 2018
Specific lncRNAs signature	One Up and five down- regulated	CAD	Unknown	Li L. et al., 2018
CHRF	Up-regulated	CH	miRNAs sponge	Wang et al., 2014
Mhrt	Down-regulated	CH	Epigenetic regulator	Han et al., 2014
CircANRIL	Up-regulation	Atherosclerosis	rRNA maturation	Holdt et al., 2016


*A brief overview of lncRNAs modulations. MI, myocardial infarction; CAD, coronary artery disease; CH, cardiac hypertrophy.*

## LncRNAs and Carbohydrate Metabolism

Most of our knowledge about the role of lncRNAs in carbohydrate control comes from studies aimed at understanding cancer energy production. Alteration of carbohydrate metabolism, in fact, is frequent in cancer because transformed cells have an enhanced glucose uptake and predominantly utilize glycolysis in order to provide to their energy needs and growth requirements, a phenomenon notoriously known as Warburg effect (Warburg et al., 1927; Bose and Le, 2018). LncRNA Ftx has been described as a regulator of peroxisome proliferator-activated receptor γ (PPARγ) pathway in hepatocellular carcinoma (HCC) (Li X. et al., 2018). Ftx is involved in lipid (Tsukahara et al., 2017) and carbohydrate metabolism (Janani and Ranjitha Kumari, 2015) and, in HCC, the level of lncRNA Ftx is significantly higher than healthy controls, enhancing glucose consumption, cell proliferation, migration and invasion (Li X. et al., 2018). Recently, analysis of lncRNA Ftx sequence through bioinformatics tools revealed that Ftx overlaps by 118 nucleotides to PPARγ gene and promoter region, suggesting that lncRNA Ftx might bind directly to PPARγ promoter enhancing transcription or acting as a PPARγ miRNA sponge (Li X. et al., 2018). Indeed, in HCC, the PPARγ pathway is more active than in non-transformed cells (or in normal tissue) (Li X. et al., 2018). This evidence suggests a mechanism of action in which a lncRNA can influence gene transcription by direct binding to a promoter region. Thus, targeting PPARγ-dependent pathway by lncRNA Ftx silencing may provide a novel approach for HCC therapy.

NBR2, a lncRNA up-regulated in cancer, is known to activate AMPK in human kidney and breast cancer cell lines, where the uptake of glucose increases in association to the activation of GLUT1 transcription (Liu X. et al., 2016). Consequently, in those cells, high intracellular glucose levels accumulate, providing a reservoir for the energy and biomass necessities of the growing tumor (Liu and Gan, 2016). In this study, in cells treated with phenformin, a drug known to up-regulate transcription of the GLUT family, NBR2 has been observed to be significantly increased. Interestingly, down-regulating NBR2, only GLUT1 resulted down-regulated, suggesting specificity for NBR2 toward a specific GLUT family member (Liu and Gan, 2016). This finding suggests that NBR2 is a phenformin-induced lncRNA that could be proposed as a target for novel anti-tumor drug design and development (Liu and Gan, 2016).

LncRNAs can modulate glycolytic enzymes such as pyruvate kinase isoform M2 (PKM2) which is highly expressed in cancer cells. In fact, in hepatocarcinoma, lncRNA H19 has been found significantly overexpressed (Li H. et al., 2015) a phenomenon paralleled by the up-regulation of PKM2 *via* transcriptional activation of the early growth response protein 1 (EGR1) (Li H. et al., 2015). Specifically, miR-675, embedded in the first exon of H19, is a repressor of HP1α that regulates EGR1, allowing the transcription of PKM2 (Li H. et al., 2015). Interestingly, PKM2 seems not only involved in glucose metabolism, but it exists into the nucleus where acts as a kinase for some transcription factors, regulating the expression of proteins involved in hypoxia resistance, proliferation, glucose uptake, and mitosis progression (Gao et al., 2012). These findings suggest that PKM2 level could be modulated through H19 lncRNA which modulation might represent a potential new anticancer strategy. In the diabetic mouse model, a recent study demonstrates the involvement of H19 in hepatocytes where it acts on FoxO1 regulation (Goyal et al., 2019). The scientists observed that in hepatic cells, FoxO1 promoter is occupied by p53 instead of H19 binding proteins which have a binding site of FoxO1 promoter sequence (Goyal et al., 2019). P53 interaction on promoter triggers the transcription of FoxO1, and as a result the activation of gluconeogenesis pathway has been reported (Goyal et al., 2019).

GAS5 is another essential tumor-associated lncRNA that, in consequence of its repression activity on the adrenocorticotropic hormone receptor, inhibits the expression of 6-phosphoglucanase (G6Pase) and phosphoenolpyruvate carboxykinase (PEPCK), resulting in a reduction of glucose storage and enhancement of glucose catabolism in cancer cells (Kino et al., 2010).

In ovarian cancer, LINC00092 lncRNA regulates phosphofructo-2-kinase/fructose-2,6-bisphosphatase 2 (PFKFB2), enhancing its expression (Zhao et al., 2017). LINC00092 expression is modulated in the response to CXCL14, a chemokine secreted by cancer-associated fibroblasts (CAFs) linked to metastasis (Zhao et al., 2017). The knockdown of LINC00092 decreases PFKFB2 expression and glucose consumption, significantly reducing metastasis. Also, the PFKFB2-induced glycolytic cancer cell phenotype seems critical for the maintenance of CXCL14 secretion from CAFs, suggesting for LINC00092 a regulatory feedback loop between cancer cells and CAFs that is important for the maintenance of the tumor microenvironment (Zhao et al., 2017).

**FIGURE 1:**
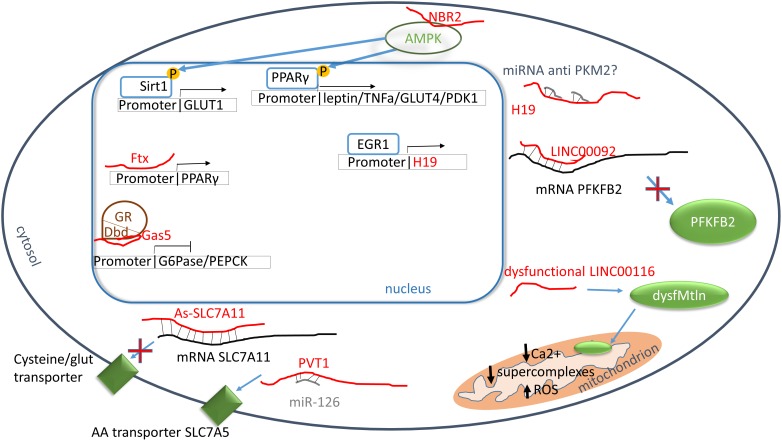
Carbohydrate and protein metabolisms. Red: lncRNAs; green: proteins; blue: transcriptor factors; brown: nuclear receptors. PPARγ promoter binds directly Ftx increasing the transcription. PPARγ is phosphorylated through AMPK pathway and it enhances the transcription of leptin, tumor necrosis factor α (TNFα), glucose channel 4 (GLUT4) and pyruvate dehydrogenase kinase 1 (PDK1). In turn, AMPK activity is enhanced by the binding to NBR2. H19 influences the transcription of pyruvate kinase M2 probably by the inhibition of PKM2 miRNAs, however, more studies are necessary to elucidate the molecular mechanism. The glucocorticoid receptor (GR) is blocked by Gas5 which interacts to GR DNA binding domain resulting in an inhibition of 6-phosphoglucanase (G6Pase) and phosphoenolpyruvate carboxykinase (PFKFB2). LINC00092 stabilizes phosphofructo-2-kinase/fructose-2,6-phosphatase 2 (PFKFB2) mRNA allowing its synthesis. LINC00116 encodes the microprotein mitoregulin (Mtln) which acts into mitochondrial inner membrane increasing the formation of supercomplexes, calcium intake and reducing ROS formation. The alteration of LINC00116 generate dysfunctional of low levels of Mtln. As-SLC7A11 binds SLC7A11 mRNA inhibiting the translation of cysteine/glutamine transporter, meanwhile plasmacytoma variant translocation 1 (PVT1) enhance the synthesis of AA transporter through the binding of miR-126 which is a suppressor of SCL7A5 mRNA.

Of particular interest for this review, in heart and skeletal muscle, the lncRNA LIN00116 has been found to encode a transmembrane microprotein, named mitoregulin, that localizes into the inner mitochondrial membrane. In consequence of the unusual coding activity of LIN00116, respiration during glucose degradation is improved, and ROS formation reduced (Stein et al., 2018) (see Table 3 and Figure 1).

**Table 3:** LncRNAs and carbohydrate metabolism regulation.

LncRNA	Expression	Disease	Role	References
Ftx	Up-regulated	HCC	Binds PPARγ promoter or miRNAs sponge	Li X. et al., 2018
NBR2	Up-regulated	Human kidney and breast cancer cell lines	Increases GLUT1 transcription	Liu and Gan, 2016
H19	Up-regulated	HCC	Increases EGR1 transcription	Li H. et al., 2015
GAS5	Up-regulated	Cancer cells	Decreases G6Pase and PEPCK transcriptions	Kino et al., 2010
LINC00092	Up-regulated	Ovarian cancer	Increases PFKFB2 transcription	Zhao et al., 2017


*HCC, hepatocellular carcinoma; PPARγ, peroxisome proliferator-activated receptor γ; GLUT1, glucose transporter 1; EGR1, early growth response protein 1; G6Pase, 6-phosphoglucanase; PEPCK, phosphoenolpyruvate carboxykinase; PFKFB2, phosphofructo-2-kinase/fructose-2,6-bisphosphatase 2.*

## LncRNAs and Their Role in Amino Acid Metabolism

A recent study demonstrates the regulation of cysteine/glutamate transporter by a lncRNA named As-SLC7A11, which is markedly reduced in epithelial ovarian cancer (Yuan et al., 2017). As a consequence of As-SLC7A11 overexpression, epithelial ovarian cancer cells were not able to use glutamate as AAs precursor or an energy source, inhibiting tumor growth (Yuan et al., 2017). Another lncRNA, the plasmacytoma variant translocation 1 (PVT1) can regulate the intake of AAs through the modulation of the miR-126/SLC7A5 axis (Li H. et al., 2018). In lung cancer, PVT1 level is increased, stimulating the uptake of AAs in cancer cells (Li H. et al., 2018). Moreover, PVT1 is induced by oxidative stress in endothelial cells, with a p53-dependent manner. Accordingly, PVT is also induced in senescent endothelial cells and decreased in PBMCs of long-living individuals (Fuschi et al., 2017) (see Table 4 and Figure 1).

**Table 4:** LncRNAs and their involvement in amino acids metabolism.

LncRNA	Expression	Disease	Role	References
AS- SLC7A11	Down-regulated	Epithelial ovarian cancer	Reduces SLC7A11 expression	Yuan et al., 2017
PVT1	Up-regulated	Lung cancer	Modulates miR-126/SLC7A5 axis	Li H. et al., 2018


*SLC7A11, cysteine/glutamate transporter; SLC7A5, amino acid transporter.*

## LncRNAs and Their Role in Lipid Metabolism

Alteration in blood lipid level is one of the most relevant risk factor in cardiovascular disease (CVD). It is well known in fact that high amount of LDL cholesterol and apolipoprotein B are associated with atherosclerotic plaques formation (Linton et al., 2000). The dangerousness of the plaque is correlated to the ease of forming vessel obstruction that cause hypoxia possibly leading to necrosis in downstream tissues causing MI or stroke (Linton et al., 2000). To prevent plaque formation, patients are often treated with drugs that inhibit the synthesis of cholesterol such as statins or more recently PCSK9 inhibitors. Cholesterol, fatty acids, and phospholipids are among the most abundant cellular lipids and their synthesis and uptake are strongly regulated, in physiological conditions, by transcription factors named sterol regulatory element binding proteins (SREBPs) (Parraga et al., 1998; Horton, 2002) which exist in three different isoforms: SREBP-1a; SREBP-1c and SREBP2 (Horton, 2002). H19 is one of the lncRNAs that modulates hepatic lipids homeostasis. It directly interacts with the polypyrimidine tract-binding protein 1 (PTBP1) enhancing its binding activity to SREBP1 that, in the presence of a high fat/high sucrose diet, translocates into the nucleus (Liu et al., 2018). Moreover, PTBP1 increases SREBP1 mRNA stability while stimulating SREBP1 cleavage (Liu et al., 2018). As a result, in hepatocytes the nuclear accumulation of SREBP1, upon H19 overexpression, seems mimicking the consequences of a fed state (Liu et al., 2018).

Other study revealed a modulation of SREBP-1c levels through the action of the lncRNA HCV regulated 1 (lncHR1) (Li D. et al., 2018). Here, in a mouse model of triglyceride accumulation, high levels of lncHR1 decreased phosphorylation and activation of the PDK1/ATK/FoxO1 pathway, inhibiting SREBP-1c expression (Li D. et al., 2018). As for many other lncRNAs, the molecular mechanism by which lncHR1 regulates SREBP-1c remains to be clarified.

MALAT1 itself can regulate lipid metabolism. In hepatic cells, recent studies demonstrated its involvement in the regulation of SREBP-1c level (Yan et al., 2016). In liver and HepG2 cells, in fact, after palmitate uptake, MALAT1 expression increase enhancing the nuclear localization of SREBP-1c that transcribes genes involved in lipid metabolism such as stearoyl CoA desaturase 1, fatty acid synthase, acetyl-CoA carboxylase 1 and ATP citrate synthase (Yan et al., 2016). Mechanistically, MALAT1 directly binds SREBP-1c, which is stabilized (Yan et al., 2016). As a result, in hepatocytes, the presence of a sufficient amount of MALAT1 decreases lipid accumulation (Yan et al., 2016).

Triglycerides metabolism needs to be strictly regulated in order to avoid the onset of metabolic syndrome and CVD. The liver-specific triglyceride regulator lncRNA Lancaster (lncLSTR) has been demonstrated to regulate triglyceride plasma levels through apolipoprotein C2 (APO-C2) and lipoprotein lipase expression (Li P. et al., 2015). Specifically, lncLSTR inhibits the farnesoid X receptor (LXR) mRNA that synthesizes for a nuclear receptor activated by bile acids and regulates APO-C2 transcription (Li P. et al., 2015). In addition, the presence of a molecular complex has been observed between lncLSTR and the TAR DNA-binding protein 43 (TDP-43) that regulates the expression of sterol 12 α hydroxylase, a key enzyme in the bile acid synthesis (Li P. et al., 2015). As a result, lncLSTR depletion improves triglycerides plasma clearance, and it could be considered as a potential new target for hypertriglyceridemia treatment.

Physiologically, levels of plasmatic lipids are regulated by apolipoproteins (APOs). It has been demonstrated that two antisense transcript lncRNAs, APOA1-AS and APOA4-AS, regulate APOA1 and APOA4 expression respectively (Halley et al., 2014; Qin et al., 2016). More in detail, APOA1-AS can modulate histone three lysine 4 (H3K4) and lysine 27 (H3K27) trimethylation (H3K4me3; H3K27me3); indeed, lncRNA silencing enhances H3K4me3 while it reduces H3K27me3 at APO gene cluster (Halley et al., 2014). Consequently, APOA1-AS knockdown increases APOs expression, enhancing lipid clearance (Halley et al., 2014). In fatty liver disease, levels of both APOA4 and APOA4-AS have been found elevated. The antisense lncRNA interacts with a stabilizing protein named HuR to stabilize APOA4 mRNA, (Qin et al., 2016). In an obese mouse model, the silencing of APOA4-AS has been associated with a reduction of cholesterol and triglycerides plasma level, suggesting this lncRNA as a potential target for new anti-hyperlipidemia drugs (Qin et al., 2016).

AT102202 is another lncRNA, which can modulate plasmatic cholesterol level inhibiting its synthesis in hepatocytes. Recently, 3-hydroxy-3-methylglutaryl-CoA reductase (HMGCR), the rate-limiting enzyme for cholesterol synthesis, has been found down-regulated by increasing levels of AT102202 (Liu et al., 2015). However, the molecular mechanism by which HMGCR is repressed remains under investigation. Remarkably, the uptake of plasmatic cholesterol it is mediated by the LDL receptor (LDLR) which is over-expressed by lncRNA RP1-13D10.2, determining a stimulation of LDL uptake and a decrease of plasma cholesterol (Mitchel et al., 2016). Indeed, RP1-13D10.2 expression increases in response to statin treatment, while in the absence of statin as well as after SREBP2 silencing, this lncRNA decreases, suggesting that RP1-13D10.2 transcription could be under control of sterol response element binding protein 2 (Mitchel et al., 2016). The molecular mechanisms involved in RP1-13D10.2 regulation of LDLR transcription are at present unknown.

**FIGURE 2:**
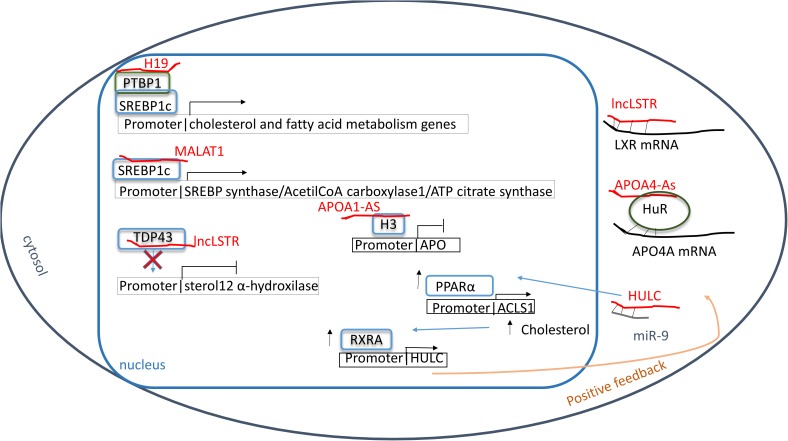
Lipid metabolism. Red: lncRNA; green: proteins; blue: transcriptional factors; gray: miRNAs. Sterol regulatory element binding protein 1c (SREBP1c) can be modulated in different ways: directly binding MALAT1 or by poly-pyrimidine tract-binding protein 1 (PTBP1) that, in turn, binds H19. As result genes involved in lipid anabolism are expressed. The long non-coding RNA lncLSTR inhibits TAR DNA-binding protein 43 (TDP-43) blocking the transcription of sterol 12 α hydroxylase and increasing the hyperlipidemia. Moreover, the hyperlipidemia is enhanced by the inhibition of APO synthesis through the chromatin condensation at APO promoters by the hypomethylation condition of histone 3 at lysine 4 and 27 due to APOA1-As bound. In addition, farnesoid X receptor (LXR) and APOA4 mRNAs are inhibited by lncLSTR and HuR protein helped by APOA4-As respectively reducing the clearance of plasmatic fatty acids. In hypercholesteremia a positive feedback has been shown in which HULC enhances PPARα synthesis. One effect is the increasing of retinoic acid X-receptor alpha (RXRA) level which recognizes and positively regulates HULC promoter.

Alteration of cholesterol metabolism is commonly found in hepatoma cells (Cui et al., 2015). Herein, the lncRNA HULC increases PPARα expression which enhances transcription of acyl-CoA synthetase long-chain family members 1 (ACSL1) (Cui et al., 2015), the enzyme appointed to long-chain fatty acid synthesis. To increase this effect, HULC down-regulates miR-9 which targets 3′UTR of PPARα transcript (Cui et al., 2015). In this context, HULC levels seem controlled by a positive feedback. Indeed, cholesterol stimulates the retinoic acid X-receptor alpha (RXRA) which recognizes and positively regulates HULC promoter (Cui et al., 2015). In a hepatoma mouse model, beneficial effects have been demonstrated by inhibition of the HULC/miR-9/PPARA/ACSL1/cholesterol/RXRA/HULC loop suggesting this feedback mechanism as a new potential therapeutic target (Cui et al., 2015). Moreover, in hepatocytes of hypercholesterolemia patients, high levels of lncRNA Activated in RCC (renal cell carcinoma) with Sunitib Resistance (lncARSR) have been found (Huang et al., 2018) where lncARSR increases the phosphorylation of AKT enhancing SREBP2 transcription (Huang et al., 2018). As a result, genes involved in cholesterol biosynthesis, such as enzymes involved in squalene synthesis including HMG-CoA reductase and HMG-CoA synthase, are up-regulated, while Cyp7a1, a limiting enzyme that converts cholesterol in bile acids, decreases (Huang et al., 2018). Also, lncARSR can also modulate SREBP-1c promoting hepatic lipogenesis. Indeed, downregulating lncARSR ameliorates lipid uptake into liver suggesting this as a new potential target for steatosis treatment (Zhang M. et al., 2018) (see Table 5 and Figure 2).

**Table 5:** LncRNAs and lipids metabolism.

LncRNA	Expression	Disease	Role	References
lncLSTR	Up-regulated	Hypertriglyceridemia	Inhibits LXR an APO-C2 transcriptions. Binds TDP-43 blocking the bile acid synthesis	Li P. et al., 2015
APOA4-AS	Up-regulated	Fatty acid liver	Stabilized HuR protein	Qin et al., 2016
APOA1-AS	Up-regulated	Hypertriglyceridemia	Modulates H3K4 and H3K27 trimethylation	Halley et al., 2014
AT102202	Up-regulated	Hypercholesterolemia	Represses HMGCR synthesis	Liu et al., 2015
HULC	Up-regulated	Hypercholesterolemia	Modulates miR-9/PPARα axis	Cui et al., 2015
LncARSR	Up-regulated	Hypercholesterolemia	Increases AKT phosphorylation	Huang et al., 2018


*LXR, farnesoid X receptor; APO-C2, apolipoprotein isoform C2; TDP-43, TAR DNA-binding protein 43; H3K4/K27, histone 3 lysine 4/27; HMGCR, 3-hydroxy-3-methylglutaryl-CoA reductase.*

## LncRNAs and Their Role in Chronic Dysmetabolic Conditions

According to the World Health Organization (WHO), in 2016 type 2 diabetes (T2D) has been the most frequently reported metabolic disease in developed countries, affecting about 8.2% of the adult population. T2D is a multifactorial disorder characterized, among other aspects, by high blood glucose and lipid levels (hyperglycemia and hyperlipidemia) in association with insulin resistance and atherosclerosis (Bornfeldt and Tabas, 2011). All these factors contribute to a condition of chronic inflammation leading to micro/macro-vascular alterations often linked to ischemia, chronic ulcers, atherosclerotic plaque formation, cardiomyopathy, amputations, and renal failure (Olefsky and Glass, 2010). Multiple epigenetic mechanisms are involved in the onset of such diabetic complications. Acetylation of histone H3 lysines 9, 14 and that of histone H4 lysine 5 (H3K9, H3K14, and H4K5) and methylation of histone H4 lysine 4 (H3K4) enriched in chromatin regions encoding for inflammatory genes correlated with the exacerbation of T2D (Reddy et al., 2015). Indeed, it has been demonstrated that in cardiac mesenchymal T2D cells the epigenetic drug pentadecylidenemalonate 1b (SPV106), an activator of histone acetylases, can restore the proliferation and differentiation potential lost in cells of cardiac origin isolated from T2D patient (Vecellio et al., 2014). Additional work reported about the presence of specific epigenetic modifications, including the increase in H3K27 tri-methylation and DNA methylation/hydroxymethylation in the chromatin and genomic DNA of cardiac fibroblasts isolated from T2D patients undergoing major cardiac surgery. In this model, a global increase of cytosine methylation has been observed in those genes involved in transcription, metabolism and glucose uptake (Spallotta et al., 2018). To revert this condition, the addition of alpha-ketoglutarate seems beneficial through its interaction with DNA demethylases (Spallotta et al., 2018).

Many lncRNAs have been associated to T2D and diabetes complications such as MALAT1, that correlates to cardiomyopathy (Bacci et al., 2018), or circRNAs targeting hsa-miR-6760-3p, hsa-miR-761, hsa-miR-6728-3p, hsa-miR-5693, hsa-miR-298, and hsa-miR-367-5p which have been correlated to depression in T2D patient (Jiang et al., 2017). However, another lncRNA, the dynamin 3 opposite strand (Dnm3os) has been recently proposed as important in T2D (Das et al., 2018). Specifically, in T2D macrophages, Dnm3os, which can bind and enhance the activity of NFκB transcription factor into the nucleus, is significantly up-regulated as compared to normal controls. As a result, Dnm3os increases the NFκB activity enhancing inflammation, phagocytosis, immune response, cell activation, chemotaxis, and response to wounding (Das et al., 2018). Moreover, Dnm3os has been found interacting with several other critical intracellular targets including nucleolin, the actin-related protein 3, ILF-2, the RNA-binding protein Ralv short isoform, as well as the heterogeneous nuclear ribonucleoprotein U like protein 2, and the variant ribonucleoprotein isoform A1 (Das et al., 2018). Interesting, nucleolin regulates chromatin structure, and the synergic effect of Dnm3os and nucleolin is increasing H3K9 acetylation at IL-6 promoter with a consequent higher inflammatory response (Das et al., 2018).

Another study indicated, in pancreatic β cells of T2D patients, the dysregulation of two lncRNAs: KQT-like subfamily member one opposite strand/antisense transcript 1 (KCNQ1OT1) and HI-LNC45 (Moran et al., 2012). The authors reported that KCNQ1OT1, abundant in cardiac tissue (Vausort et al., 2014), was found over-expressed in diabetic pancreatic cells while HI-LNC45 was significantly lower than in healthy controls (Moran et al., 2012). Despite this evidence, their functional role in diabetes remains unclear.

In addition to its role in cancer and CVD, MALAT1 is also significantly up-regulated in diabetic gastroparesis (DGP) (Gong et al., 2018), which is a clinically relevant complication of diabetes mellitus characterized by bloating, nausea and vomiting (Kashyap and Farrugia, 2010). In DGP rat models and gastric smooth muscle cells isolated from DGP patients, the accumulation of MALAT1 has been associated with the decrease of MHC and alpha-smooth muscle actin (αSMA), which are proteins involved in contractile and adhesion functions (Gong et al., 2018), resulting in a chronic smooth muscle cell enlargement which contributes to symptoms. This study shows that high glucose increases MALAT1 level and that after MALAT1 silencing contractile markers are re-expressed reducing DGP exacerbation at least in diabetic mice (Gong et al., 2018). Although in this condition, MALAT1-dependent mechanism of action has not been elucidated yet, these findings suggest MALAT1 as a potential target for the development of new DGP therapeutic strategies.

Diabetic cardiomyopathy is a critical complication of T2D (Jia et al., 2018). Interestingly, upon prolonged exposure to elevated blood glucose, high level of MALAT1 has been found in mouse cardiomyocytes (Bacci et al., 2018). Sildenafil, the pharmacological inhibitor of phosphodiesterase 5 (PD5), which increases endogenously cardiac and vascular levels of nitric oxide, normalized MALAT1 expression with beneficial effects in an experimental mouse model of diabetic cardiomyopathy (Bacci et al., 2018).

Remarkably, lncRNAs can also act as hormone-like molecules. In fact, fluctuating circulating levels have been detected body fluids such as peripheral blood (Shen et al., 2017) and urine (Terracciano et al., 2017) and lncRNA-p3134 secreted by defective β-cells and circulating in exosomal vesicles, has been found highly expressed in T2D patients’ plasma samples (Ruan et al., 2018) where lncRNA-p3134 expression levels increased proportionally to blood glucose (Ruan et al., 2018). Interestingly, this lncRNA has been shown to increase insulin synthesis in healthy β cells, suggesting a role as an epigenetic regulatory element. Indeed, normal β cells reacted to lncRNA-p3134 reducing caspase 3 and 9 and Bcl2 levels, thus protecting themselves from glucotoxicity (Ruan et al., 2018). Inhibiting PI3K/AKT axis, the protective effect of lncRNA-p3134 was significantly attenuated (Ruan et al., 2018). Despite these findings, the correlation between lncRNA-p3134 regulation/function and insulin signaling remains mostly unclear.

In T2D patients, many epigenetic alterations have been found in non-pancreatic cells. A recent study reported that lncRNA metallothionein one pseudogene 3 (MT1P3) is up-regulated in T2D megakaryocytes (Zhou et al., 2018). MT1P3 regulates the expression of the p2y12 receptor by sponging out miR-126 that targets p2y12 mRNA (Zhou et al., 2018). Also, MT1P3 enhances the p2y12 presence in platelet membranes increasing the normal activity of the receptor (Zhou et al., 2018). P2y12 is a G-coupled receptor, and it is activated by ADP stimulating platelets aggregation (Hu et al., 2017). Clinically, in T2D patients that frequently fail to respond to antiplatelet therapy (Samos et al., 2016), the alteration of the MT1P3 epigenetic pathway seems associated with risk of microcirculation complications and thrombotic microangiopathies higher than in the non-diabetic population (Ding et al., 2017). In this condition, a myocardial re-infarction can occur after an episode of in-stent thrombosis while standard clopidogrel treatment results ineffective (Samos et al., 2014).

One of the mechanisms of action accredited to lncRNAs is binding and regulating transcription factors. An example in this direction is given by lncRNA Meg3 which modulates the musculoaponeurotic fibrosarcoma protein A (MafA) in mouse β cells through interaction with EZH2 (Wang N et al., 2018), one of the methyl-transferases of Polycomb complex (Cao et al., 2002). EZH2 represses MafA inhibitors possibly present in specific genomic regions, allowing the transcription factor to be active (Wang N et al., 2018). In fact, in diabetic mice, Meg3 down-regulation is paralleled by a decrease of MafA with the consequent reduction in insulin synthesis and secretion (Zhang et al., 2005).

In diabetic retinopathy, a severe and common complication of diabetes, aberrant expression of several lncRNAs has been frequently reported (Yan et al., 2014). Here, those lncRNAs involved in MAPK and chemokine signaling pathways, axon guidance, complement, and coagulation cascades and pyruvate metabolism were found up-regulated (Yan et al., 2014). All these alterations are known associated with cellular stress response, DNA repair, epithelium and tube development and lens formation (Yan et al., 2014). Specifically, it has been discovered that MALAT1 is upregulated in retina being present in the aqueous humor of diabetic mice and patients. This result suggests for MALAT1 potential application as a new biomarker of retinopathy (Yan et al., 2014).

Novel findings indicate that lncRNA H19 is highly expressed in diabetic liver (Zhang N. et al., 2018). Here, H19 has been found enriched into the nucleolus and able to regulate gluconeogenesis *via* DNA methylation control at the level of transcription factor hepatocyte nuclear factor 4 alpha (HNF4A) which transcribes glucose-6-phosphatase (G6PC) and phosphoenolpyruvate carboxykinase 1 (PCK1) (Zhang N. et al., 2018). In hyperglycemia, high levels of H19 in the liver contribute to HNF4A promoter demethylation which results repressed at transcriptional level inhibiting G6PC and PCK1 synthesis essential for glycogen formation and storage (Zhang N. et al., 2018). As a consequence, H19 overexpression promotes hepatic glucose production (Zhang N. et al., 2018) and insulin resistance (Gabory et al., 2010; Zhang N. et al., 2018). Moreover, it has been demonstrated that in hepatic cells H19 binds a set of DNA-binding proteins associated to FoxO1 promoter that inhibit FoxO1 transcription resulting in a downregulation of gluconeogenesis pathway in healthy cells (Goyal et al., 2019). To simulate the diabetic conditions, H19 has been silenced in mouse hepatocytes and as results on FoxO1 promoter p53 has been found associated activating the cascade that exacerbates in insulin resistance (Goyal et al., 2019).

During fasting, the hepatic glucokinase (CGK), another enzyme involved in glycogen synthesis and storage, is physiologically downregulated (Massa et al., 2011). This phenomenon occurs at, least partially, in consequence of the up-regulation of lncRNA liver CGK repressor (lncLGR) (Ruan et al., 2016). It has been demonstrated that during fasting, lncLGR is overexpressed binding to heterogeneous nuclear ribonucleoprotein L (hnRNPL) which is recruited to CGK gene promoter repressing its transcription (Ruan et al., 2016). As a consequence, glycogen is not stored remaining accessible for glucose synthesis (Ruan et al., 2016).

As in CVD, a SNP in ANRIL gene has been found to correlate with T2D exacerbation. Patients who carry rs10811661 (Broadbent et al., 2008) or rs564398 (Kong et al., 2018) have more risk to develop diabetes. Especially, rs564398 causes T2D by the reduction of b-cell proliferation (Kong et al., 2018). All these findings in ANRIL locus suggest that a lncRNA polymorphism can be a new risk factor of non-transmissible diseases. An exciting discovery would be if the lncRNA and its variants could be detected in peripheral blood and used as a diagnostic biomarker.

Another condition, in which the metabolism results altered, is obesity that is defined through the valuation of body mass index (BMI, kg/m^2^). Conventionally, the cut off between overweight and obese is a BMI higher than 30. The clinical picture of obesity is very complex due to the condition, called metabolic syndrome, in which insulin resistance, glucose intolerance, hypertension, and dyslipidemia coexist (Reaven, 2005).

Moreover, the obesity is a risk factor for many diseases such as CVD, non-alcoholic steatohepatitis (NASH), polycystic ovarian syndrome (PCOS), obstructive sleep apnea (OSA), and cancers (Einhorn et al., 2003). Recently, in obese mouse models, it has been discovered a lncRNA (lncSHGL) which is significantly downregulated in hepatocytes while in obese human a homologous (B4GALT1-AS1) results decreased in hepatocytes too (Wang J. et al., 2018). This study reveals the physiological lncSHGL ability to recruits heterogeneous nuclear ribonucleoprotein A1 (hnRNPA1) that increases the efficiency of the translation of calmodulin (CaM) mRNA without altering the transcription (Wang J. et al., 2018). As a result, in healthy hepatocytes, PI3K/Akt pathway is activated while mTOR/SREPB pathway is inhibited and hepatic gluconeogenesis and lipogenesis are suppressed in an insulin-independent way (Wang J. et al., 2018). In conclusion, low levels of B4GALT1-AS1 in obese patients cause a continuous activation of the lipid synthesis determining their accumulation (Wang J. et al., 2018) (see Table 6).

**Table 6:** LncRNAs in dysmetabolic conditions.

LncRNA	Expression	Disease	Role	References
MALAT1	Up-regulated	Cardiomyopathy in T2D patients	Unknown	Bacci et al., 2018
	Up-regulated	Gastroparesis in T2D patients	Decreases the transcription of MHC and αSMA	Gong et al., 2018
	Up-regulated	Retinopathy in T2D patients	Unknown	Yan et al., 2014
hsa-miR-6760-3p, hsa-miR-761, hsa-miR-6728-3p, hsa-miR-5693, hsa-miR-298, hsa-miR-367-5p	Up-regulated	Depression in T2D patients	Unknown	Jiang et al., 2017
Dmn3os	Up-regulated	T2D	Enhances the activity of NFκB. Binds nucleolin, to enhance H3K9 acetylation	Das et al., 2018
KCNQ1OT1	Up-regulated	T2D	Unknown	Moran et al., 2012
HI-LNC45	Down-regulated	T2D	Unknown	Moran et al., 2012
lncRNA-p3134	Up-regulated	Peripheral blood in T2D patient	Hormone-like	Ruan et al., 2018
MT1P3	Up-regulated	T2D	Modulates miR-126/p2y12 axis	Zhou et al., 2018
H19	Up-regulated	T2D	Modulates DNA demethylation of gluconeogenesis gene promoters	Zhang N. et al., 2018
LncLGR	Up-regulated	Fasting condition	Binds hnRNPL repressing glycogen storage	Ruan et al., 2015


*T2D, type 2 diabetes; MHC, myosin heavy chain; αSMA, α-smooth muscle actin; hnRNPL, heterogeneous nuclear ribonucleoprotein L.*

## Conclusion

Recent discoveries in epigenetics enlightened important functional and pathophysiological roles for lncRNAs. Dysregulation of this component of our transcriptome, in fact, has been found in pathological condition such as cancer (Terracciano et al., 2017; Chen M. et al., 2018; Ma et al., 2018; Tang et al., 2018), CVD (Thum and Condorelli, 2015; Mitchel et al., 2016; Viereck and Thum, 2017), and dysmetabolic conditions including obesity and T2D (Moran et al., 2012; Yan et al., 2016; Das et al., 2018). Although lncRNAs molecular mechanism of action is far to be clarified, it seems clear that they can act as miRNA sponges (Chen L. et al., 2018; Zhao et al., 2018) or directly binding regulatory proteins such as transcription factors, nuclear hormone receptors (Aiello et al., 2016) or epigenetic enzymes (Khalil et al., 2009), thus controlling gene expression and protein function. Remarkably, lncRNAs have also a pivotal role in carbohydrate and lipid metabolism, and their alteration greatly influences cellular homeostasis (Liu et al., 2018) contributing, for instance, to insulin-resistance (Yan et al., 2016) or formation of atherosclerotic plaques (Huang et al., 2018). In light of this evidence, although our mechanistic knowledge is still limited, lncRNAs seem to play an enormously crucial biological role regulating cellular metabolism at multiple levels, in physiological as well as pathophysiological conditions. This fact is unquestionable and may open essential avenues for the future development of novel therapeutic strategies in chronic diseases.

## Author Contributions

All authors have contributed to the conception and design of the article and revision. AM and CG wrote the text. AF and FM provided the suggestions.

## Conflict of Interest Statement

The authors declare that the research was conducted in the absence of any commercial or financial relationships that could be construed as a potential conflict of interest.
